# The Unseen Toll: Impact of COVID-19 on Professional Quality of Life of Healthcare Workers

**DOI:** 10.7759/cureus.88633

**Published:** 2025-07-23

**Authors:** Hemlata B Munjappa, Meena K Parekh, Aditi N Patil, Smita A Shinde, Sunita S Ingale

**Affiliations:** 1 Physiology, Bharati Vidyapeeth (Deemed to be University) Medical College and Hospital, Sangli, IND; 2 Pharmacology, Bharati Vidyapeeth (Deemed to be University) Medical College and Hospital, Sangli, IND

**Keywords:** burnout, compassion satisfaction, covid-19 pandemic, : healthcare workers, professional quality of life

## Abstract

Background

During the COVID-19 pandemic, healthcare workers (HCWs) were at risk of getting affected emotionally, physically, and psychologically. Repeated exposure to traumatic situations, concern for patients' well-being, social distancing measures, quarantine, and stigma lead to feelings of loneliness and depression. Stress, anxiety, insomnia, and burnout syndrome were the common psychological consequences noted in HCWs during the COVID-19 pandemic. During this pandemic period, improvements in positive patient outcomes and expressions of gratitude towards healthcare workers provided positive reinforcement for them. This positive feeling buffered the feeling of burnout. The balance between these two explains the professional quality of life (ProQOL). So this study aimed to study the professional quality of life of healthcare workers involved during the COVID-19 pandemic at a tertiary healthcare center.

Material and methodology

The study was conducted by using a pre-validated and reliable ProQOL questionnaire to assess the professional quality of life in 161 HCWs from a tertiary healthcare center (62 doctors, 89 nurses, and 10 technicians).

Results

ProQOL has positive (compassion satisfaction) and negative (compassion fatigue and burnout) aspects that individuals feel during their work as a helper. Compassion Satisfaction, Burnout, and Secondary Traumatic Stress were moderate in all three groups. Moderate level of Compassion Satisfaction was seen in 57 (91.93%) of doctors, 78 (87.6%) of nurses and 4 (40%) of technicians. Moderate level of Burnout was seen in 55 (88%) of doctors, 81 (91%) of nurses and 6 (60%) of technicians. Moderate level of Secondary Traumatic Stress was seen in 56 (90%) of doctors, 72 (80%) of nurses and 7 (70%) of technicians.

Conclusion

Overall, the moderate scores across all dimensions highlight the delicate balance maintained by healthcare workers between professional fulfillment and occupational stress during the pandemic. Institutional support systems, mental health interventions, peer support programs, and adequate staffing will promote a healthier professional quality of life among HCWs in high-stress environments like pandemics.

## Introduction

The COVID-19 pandemic has placed a huge burden on healthcare systems worldwide, and the healthcare workers (HCW) are exposed to the maximum risk of getting infected by SARS-CoV-2 [1]. The emotional, physical, and psychological effect of the COVID-19 pandemic on HCWs was substantial, as they were the front-line warriors. High fear of getting infection, long working hours, lack of rest, sleep deprivation, and inadequate supply of personal protective equipment (PPE) kits lead to stress and anxiety development in HCWs [2-5]. Repeated exposure to traumatic situations, concern for patients' well-being, social distancing measures, quarantine, and stigma lead to feelings of loneliness and depression. HCWs had faced decreased job satisfaction, increased intention to leave the profession, and concerns about their future career [6, 7]. We should not ignore the pandemic's effect on the mental health of HCWs [8].

Professionals who were involved in the care of COVID-19 patients had work-related psychological pressure and frequent somatic symptoms [9]. During the COVID-19 pandemic, it has been noted that the sleep duration was insufficient and the quality unsatisfactory, in addition to the decreased physical and mental functioning during the day in medical professionals [10]. Secondary traumatic stress, anxiety, and burnout syndrome were the common psychological consequences noted in HCWs during the COVID-19 pandemic [11-13].

Along with negative consequences, the COVID-19 pandemic brought some positive changes in society that should be analyzed. The people started expressing gratitude and closeness to HCWs. During this period, the HCWs were in the spotlight, and these developments helped them to get positive reinforcement. Some of them hypothesized that these positive things buffered the feeling of burnout during the pandemic and gave the sense of self-efficacy to the HCWs [14, 15].

As described earlier, these difficult circumstances created the new complex working environment [16]. During the pandemic period, work-related stress manifested as increased health concerns in society [8, 17-19]. Unpredictable exposure to infection with long working hours created stress, anxiety, and exhaustion, leading to feelings of compassion fatigue, but on the other hand, with improvement in positive patient outcomes, society started admiring HCWs without historical precedents and improved public opinion [16, 20].

Professional quality of life (ProQOL) has a positive aspect that is compassion satisfaction (CS) and a negative aspect that is compassion fatigue (CF) that individuals feel during their work as a helper [21]. Compassion satisfaction has been described as the pleasure or positivity derived from caregiving, which also includes satisfaction from helping co-workers and feeling that their work has social value [22]. Compassion fatigue, or STS, has been described as the negative effects that result from stress experienced through a traumatizing event [23]. CF has been called the cost of caring for clients or their emotional pain that is caused by stress from helping people and the desire to relieve their suffering [20]. The balance between CS and CF within the workplace shows the level of ProQOL [23, 24]. CS and CF have been seen among health care professionals at the time of individual, community, national, and even worldwide crises [22].

So, this study aimed to compare the professional quality of life (ProQOL), including compassion satisfaction, burnout, and secondary traumatic stress among healthcare workers (doctors vs nurses vs technicians) at a tertiary care hospital during the second wave of the COVID-19 pandemic.

## Materials and methods

Study design

The study was a single-center, cross-sectional, self-reported questionnaire survey conducted at Bharati Vidyapeeth (Deemed to be University) Medical College and Hospital, Sangli, which is a tertiary healthcare facility, following approval from the Institutional Ethics Committee (Approval No. BV(DU)MC&H/Sangli/IEC/435/21). The study was conducted during the year 2021-22 for one year.

Study participants

Participants included doctors, nurses, and technicians providing care to COVID-19-positive patients at the center. Both male and female healthcare workers (HCWs) aged between 20 and 60 years were invited to participate, and informed consent was obtained. Participants were assured that data would be kept confidential and it was mentioned accordingly in the consent form. HCWs with any known medical conditions such as hypertension, diabetes mellitus, endocrine disorders affecting sleep, sleep disorders, or psychiatric conditions were excluded based on self-reported history.

Study procedure

The survey was conducted online using a Google Form, which was distributed to participants via WhatsApp. We periodically sent reminder messages through the participants' WhatsApp group. Only fully completed questionnaires were included in the final analysis. A total of 175 participants were invited for the study; only 161 complete responses were received. Sociodemographic data, including participants’ age, sex, and marital status, were collected.

Tool

To assess the professional quality of life of healthcare workers during the COVID-19 pandemic, the Professional Quality of Life Scale: Compassion Satisfaction and Fatigue Version 5 (ProQOL V), developed by B. Hudnall Stamm, was used [21].

The ProQOL scale explores the positive and negative impacts of helping others, as healthcare workers’ compassion can influence well-being. ProQOL integrates two main dimensions: Compassion Satisfaction (CS) and Compassion Fatigue (CF). Compassion Fatigue includes Burnout (characterized by exhaustion, frustration, anger, and depression) and Secondary Traumatization (ST). The internal consistency of the scales, measured by Cronbach’s alpha, was reported as 0.88 for CS, 0.75 for Burnout, and 0.81 for ST.

All three subscales had 10 questions each, and were categorized into mild, moderate and severe levels depending on the score. The scale was assessed according to the 5-point Likert scale with 1 - Never and 5 - Very Often. Participants were asked to reflect honestly on how frequently they experienced various feelings or situations in the past 30 days, selecting responses on a Likert scale ranging from 1 (never) to 5 (very often). The raw scores for each category range from 10 to 50, with established score ranges for interpretation. If the sum of scores from the questions asked comes 22 or less, then the level is mild; if the score comes between 23 to 41, then the level will be moderate and if the score is more than 42, then it is severe.

Statistical analysis

The collected data were compiled and analyzed using SPSS version 29.0 (IBM Corp., Armonk, NY, USA). Percentage was calculated by using Microsoft Excel (Microsoft Corp., Redmond, WA, USA). The Chi-square (χ²) test was applied to assess the association between different categorical variables. A p-value of <0.05 was considered statistically significant.

## Results

Data was collected from 161 participants working as healthcare providers at a tertiary health care center. There were 62 doctors, 89 nurses and 10 technicians who were serving the COVID-19 patients.

Out of 161 HCWs, 85 (52.79%) were male and 76 (47.20%) were female participants. The number of participants in the 20-30 years age group was 45 (27.9%), 30-40 years was 48 (29.81%), 40-50 years was 49 (30.43%) and 50-60 years was 19 (11.80%). Married HCWs were 109 (67.70%) while non-married were 52 (32.29%) (Table 1).

**Table 1:** Sociodemographic factors of healthcare workers

Sociodemographic Factors		Doctors	Nurses	Technician	Total
Sex	Male	35(41.17% )	44(51.76%)	6(7.05%)	85 (52.79%)
	Female	27(35.52%)	45(59.21%)	4(5.26%)	76 (47.20%)
Age	20-30	19(42.22%)	23(51.11%)	3(6.66%)	45(27.95%)
	30-40	15(31.25%)	29(60.41%)	4(8.33%)	48(29.81%)
	40-50	22(44.89%)	24(48.97%)	3(6.12%)	49(30.43%)
	50-60	6(31.57%)	13(68.42%)	0	19(11.80%)
Married	Yes	38(34.86%)	67(61.46%)	4(3.66%)	109(67.70%)
	No	24(46.15%)	22(42.30%)	6(11.53%)	52(32.29%)

Moderate level of compassion satisfaction was seen in 57 (91.93%) of doctors, 78 (87.6%) of nurses and 4 (40%) of technicians. Higher scores of moderate level of compassion satisfaction were found to be related to doctors, whereas lower scores of moderate level of compassion satisfaction were found with technicians. Mild compassion satisfaction was seen in 4 (6.45%) of doctors, 9 (11.25%) of nurses and 3 (30%) of technicians, and severe was seen in 1 (1.61%) of doctors, 2 (2.24%) of nurses and 3 (30%) of technicians. Compassion satisfaction scores are significantly dependent on healthcare workers (Table 2).

**Table 2:** Comparison of Compassion Satisfaction Score among healthcare workers

Healthcare Workers	Compassion Satisfaction score (CS)				Chi-square statistic	p-value
	Mild	Moderate	Severe	Total	27.3294	0.000017
Doctor	4 (6.45%)	57 (91.93%)	1 (1.61%)	62		
Nurses	9 (11.25%)	78 (87.6%)	2 (2.24%)	89		
Technician	3 (30%)	4 (40%)	3 (30%)	10		
TOTAL	16 (10%)	139 (86%)	6 (3.7%)	161		

Moderate level of Burnout was seen in 54 (88%) of doctors, 81 (91%) of nurses and 6 (60%) of technicians. Mild Burnout was seen in 5 (8%) of doctors, 5 (5.6%) of nurses and 3 (30%) of technicians, and severe was seen in 3 (4.8%) of doctors, 3 (3%) of nurses and 1 (10%) of technicians. Burnout scores are not dependent on healthcare workers; however, higher scores of moderate level of burnout were seen in nurses and lower scores of moderate level of burnout are related to technicians (Table 3).

**Table 3:** Comparison of Burnout Score among healthcare workers

Healthcare Workers	Burnout score				Chi-square statistic	p-value
	Mild	Moderate	Severe	Total	8.5732	0.0727
Doctor	5 (8%)	54 (88%)	3 (4.8%)	62		
Nurses	5 (5.6%)	81 (91%)	3 (3%)	89		
Technician	3 (30%)	6 (60%)	1 (10%)	10		
TOTAL	13 (8%)	141 (88%)	7 (4%)	161		

Moderate level of secondary traumatic stress was seen in 56 (90%) of doctors, 72 (80%) of nurses and 7 (70%) of technicians. Higher scores of moderate level of secondary traumatic stress were related to doctors. Mild secondary traumatic stress was seen in 5 (8%) of doctors, 9 (10%) of nurses and 2 (20%) of technicians, and severe was seen in 1 (2%) of doctors, 8 (9%) of nurses and 1 (10%) of technicians (Table 4).

**Table 4:** Comparison of Secondary Traumatic Stress among healthcare workers

Healthcare Workers	Secondary Traumatic Stress Score				Chi-square statistic	p-value
	Mild	Moderate	Severe	Total	5.3186	0.256137
Doctor	5 (8%)	56 (90%)	1 (2%)	62		
Nurses	9 (10%)	72 (80%)	8 (9%)	89		
Technician	2 (20%)	7 (70%)	1 (10%)	10		
TOTAL	16 (10%)	135 (83.8%)	10 (6.2%)	161		

## Discussion

The present study evaluated the professional quality of life among healthcare personnel, which included doctors, nurses and technicians working in a tertiary care hospital during the second wave of the COVID-19 pandemic, finding moderate levels of compassion satisfaction, burnout and secondary traumatic stress. These results reflect both the resilience and psychological strain experienced by healthcare workers during a period of heightened health crisis.

The moderate levels of compassion satisfaction observed are consistent with findings by Ruiz-Fernández et al. (2021) and Trumello et al. (2020) who reported that healthcare professionals continued to derive meaningful satisfaction from their roles despite the adversities of the pandemic [25,26]. Compassion satisfaction is a critical protective factor that can mitigate the negative effects of occupational stress, although the moderate scores here suggest that the ongoing pressures may have reduced the ability of healthcare personnel to fully experience professional fulfilment [27,28].

Regarding burnout, the moderate levels align with a study conducted by Lai et al. (2020), where healthcare workers faced emotional exhaustion, depersonalization and reduced personal accomplishment during the pandemic [5]. Although the scores in the present study are not in the high-risk range, the persistence of moderate burnout levels is concerning, particularly given the prolonged nature of the COVID-19 crisis. Similar trends were identified in studies from Taiwan and Iran [29,30], emphasizing the global impact of the pandemic on healthcare worker well-being. Moderate levels of secondary traumatic stress were also noted. Secondary traumatic stress results from indirect exposure to patients’ trauma and suffering and can lead to significant psychological distress if unaddressed. Ruiz-Fernández et al. (2020) and Serrão et al. both highlighted the risk of secondary traumatic stress in healthcare workers during COVID-19, emphasizing the need for early interventions [20,31]. Similarly, Cuartero-Castañer et al. (2021) emphasized that healthcare personnel dealing with critically ill patients are particularly vulnerable to developing symptoms akin to post-traumatic stress disorder [32].

The findings of this study also suggest that even outside of pandemic conditions, healthcare workers are at inherent risk for professional burnout and secondary stress, particularly in high-demand settings such as tertiary care hospitals.

Overall, the moderate scores across all dimensions highlight the delicate balance maintained by healthcare workers between professional fulfillment and occupational stress during the pandemic. Institutional support systems, mental health interventions, peer support programs and adequate staffing are critical to sustaining the well-being of healthcare personnel. Longitudinal studies are recommended to assess the evolution of professional quality of life over time and to better understand protective factors that can enhance resilience among healthcare workers.

Although the number of participants included in the present study provided a sufficient sample size for evaluations, studies in technicians with larger sample size will further contribute to the research domain. The results are based on self-report, which might have resulted in underreporting sensitive information including symptoms of STS and/or burnout. As this is a single-center study, to generalize the results, the study could have been done as a multicentric study. It is a cross-sectional design, so it could not assess causality or changes over time. Adjustment for confounders (e.g., shift timing, previous trauma, COVID-19 exposure level) was not done, which may impact ProQOL scores.

## Conclusions

The study highlights that healthcare workers (HCWs) during the COVID-19 pandemic experienced a moderate level of compassion satisfaction, burnout, and secondary traumatic stress, reflecting a complex interplay between the emotional rewards and challenges of their profession. The scores of moderate level compassion satisfaction and secondary traumatic stress were higher in doctors, while that of burnout was higher in nurses. Compassion satisfaction scores were significantly dependent on healthcare workers. Despite facing significant psychological stressors, the positive reinforcement from improved patient outcomes and public appreciation provided a buffering effect against burnout. These findings underscore the importance of ongoing support systems and interventions to enhance compassion satisfaction and mitigate compassion fatigue, thereby promoting a healthier professional quality of life among HCWs in high-stress environments like pandemics.

## Appendices

PROFESSIONAL QUALITY OF LIFE SCALE (PROQOL)

COMPASSION SATISFACTION AND COMPASSION FATIGUE (PROQOL) VERSION 5 (2009)

When you [help] people, you have direct contact with their lives. As you may have found, your compassion for those you [help] can affect you in positive and negative ways. Below are some questions about your experiences, both positive and negative, as a [helper]. Consider each of the following questions about you and your current work situation. Select the number that honestly reflects how frequently you experienced these things in the last 30 days.           

**Table 5:** The Professional Quality of Life Scale: Compassion Satisfaction and Fatigue Version 5 (ProQOL V)

Sr. No.	Questions	5 Point LIKERT Scale				
		1=Never	2=Rarely	3=Sometimes	4=Often	5=Very Often
1	I am happy.	-	-	-	-	-
2	I am preoccupied with more than one person I [help].	-	-	-	-	-
3	I get satisfaction from being able to [help]people.	-	-	-	-	-
4	I feel connected to others.	-	-	-	-	-
5	I jump or am startled by unexpected sounds.	-	-	-	-	-
6	I feel invigorated after working with those I [help].	-	-	-	-	-
7	I find it difficult to separate my personal life from my life as a [helper].	-	-	-	-	-
8	I am not as productive at work because I am losing sleep over traumatic experiences of a person I	-	-	-	-	-
9	I think that I might have been affected by the traumatic stress of those I [help].	-	-	-	-	-
10	I feel trapped by my job as a [helper].	-	-	-	-	-
11	Because of my [helping], I have felt "on edge" about various things.	-	-	-	-	-
12	I like my work as a [helper].	-	-	-	-	-
13	I feel depressed because of the traumatic experiences of the people I [help].	-	-	-	-	-
14	I feel as though I am experiencing the trauma of someone I have [helped].	-	-	-	-	-
15	I have beliefs that sustain me.	-	-	-	-	-
16	I am pleased with how I am able to keep up with [helping] techniques and protocols.	-	-	-	-	-
17	I am the person I always wanted to be.	-	-	-	-	-
18	My work makes me feel satisfied.	-	-	-	-	-
19	I feel worn out because of my work as a [helper].	-	-	-	-	-
20	I have happy thoughts and feelings about those I [help] and how I could help them.	-	-	-	-	-
21	I feel overwhelmed because my case [work] load seems endless.	-	-	-	-	-
22	I believe I can make a difference through my work.	-	-	-	-	-
23	I avoid certain activities or situations because they remind me of frightening experiences of the people I [help].	-	-	-	-	-
24	I am proud of what I can do to [help].	-	-	-	-	-
25	As a result of my [helping], I have intrusive, frightening thoughts.	-	-	-	-	-
26	I feel "bogged down" by the system.	-	-	-	-	-
27	I have thoughts that I am a "success" as a [helper].	-	-	-	-	-
28	I can't recall important parts of my work with trauma victims.	-	-	-	-	-
29	I am a very caring person.	-	-	-	-	-
30	I am happy that I chose to do this work.	-	-	-	-	-
